# A randomized controlled trial of pectoralis major myofascial release massage for breastfeeding mothers: breast pain, engorgement, and newborns’ breast milk intake and sleeping patterns

**DOI:** 10.4069/kjwhn.2023.03.15

**Published:** 2023-03-31

**Authors:** Won-Ryung Choi, Yeon-Suk Kim, Ju-Ri Kim, Myung-Haeng Hur

**Affiliations:** 1 Sanbon Branch of Mamslibe, Gunpo, Korea; 2 Department of Nursing, Chung Cheong University, Cheongju, Korea; 3 Department of Nursing, Kyung Min University, Uijeongbu, Korea; 4 College of Nursing, Eulji University, Uijeongbu, Korea

**Keywords:** Breast feeding, Human milk, Massage, Pain, Pectoralis muscles

## Abstract

**Purpose:**

Supportive interventions to improve breastfeeding practice are needed in nursing. This study investigated the effects of pectoralis major myofascial release massage (MRM) on breast pain and engorgement among breastfeeding mothers and on breast milk intake and sleep patterns among newborns.

**Methods:**

Breastfeeding mothers who had delivered between 37 and 43 weeks and had 7- to 14-day-old newborns were recruited from a postpartum care center in Gunpo, Korea. Participants were randomized to the MRM or control group. The outcome variables were breast pain and breast engorgement among breastfeeding mothers and breast milk intake and sleep time among newborns. The experimental treatment involved applying MRM to separate the pectoralis major muscle and the underlying breast tissue in the chest. After delivery, the first MRM session (MRM I) was provided by a breast specialist nurse, and the second (MRM II) was administered 48 hours after MRM I.

**Results:**

Following MRM, breast pain (MRM I: t=−5.38, *p*<.001; MRM II: t=−10.05, *p*<.001), breast engorgement (MRM I: right, t=−1.68, *p*=.100; left, t=−2.13, *p*=.037 and MRM II: right, t=−4.50, *p*<.001; left, t=−3.74, *p*<.001), and newborn breast milk intake (MRM I: t=3.10, *p*=.003; MRM II: t=3.09, *p*=.003) differed significantly between the groups.

**Conclusion:**

MRM effectively reduced breast engorgement and breast pain in breastfeeding mothers, reducing the need for formula supplementation, and increasing newborns’ breast milk intake. Therefore, MRM can be utilized as an effective nursing intervention to alleviate discomfort during breastfeeding and to improve the rate of breastfeeding practice (clinical trial number: KCT0002436).

## Introduction

Breast milk is an important nutrient source for both preterm and term infants, as reported in numerous studies [1]. Breast milk has several benefits: it improves infant digestion, lowers morbidity rates, reduces atopy by minimizing exposure to allergens, and stimulates the development of an emotionally stable personality [2]. For mothers, uterine contractions help prevent postpartum bleeding and reduce the severity of anemia [3]. In addition, breastfeeding assists with weight loss by burning calories [4]; lowers the risks of premenopausal breast cancer, ovarian cancer, and osteoporosis; and ultimately helps establish an intimate bond between mother and child [5,6].

In South Korea (hereinafter, Korea), the breastfeeding rate was 90.0% in the 1970s; however, this rate has progressively declined despite the advantages of breastfeeding [7]. Thus, one goal of Korea’s Fourth National Health Promotion Policy was to increase the rate of breastfeeding continuation for 6 months to 66.8% by 2020 [8]. The rate of breastfeeding in Korea is high (95.6%) immediately after birth, but drops to 47.5% by 3 months of age, and fewer than two in 10 mothers continue to breastfeed for 6 months [9]. This information suggests that despite its many advantages, breastfeeding is associated with various problems [3]. These include discomforts such as breast pain, breast engorgement, nipple damage, lack of breast milk, fatigue from frequent feeding, and lack of sleep, which inhibit breastfeeding [10-12]. Due to these factors, alternative food consumption (breast milk with formula feeding, or formula feeding alone) has become increasingly common in Korea [13].

**Figure 1.**
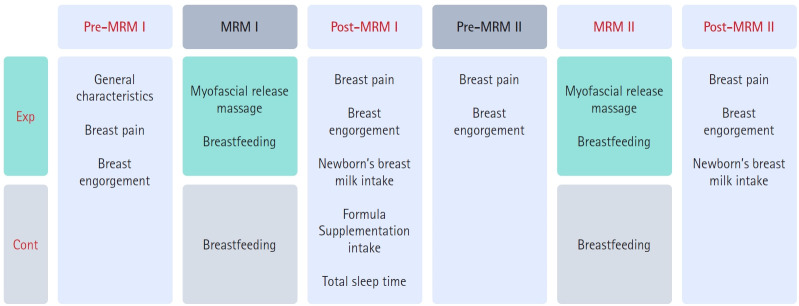
Study design. Cont: Control group; Exp: experimental group; MRM: myofascial release massage; MRM I: the first pectoralis major MRM; MRM II: second pectoralis major MRM (performed 48 hours after MRM I).

With recognition of the need for preventive health care to reduce discomfort and difficulty during breastfeeding [12], nursing interventions such as cabbage therapy, breast massages, and education on the benefits of breast milk have been provided to mothers at the commencement of prenatal care. Breast massage has been found effective in alleviating breast discomfort [14]. Specifically, pectoralis major myofascial release massage (MRM) involves gently separating the firm connective tissue between the mammary gland and the pectoralis major muscle using hand pressure and movement to create space at the rear of the breast tissue. This improves blood circulation, softens the hard breast tissue, and reduces pain [15]. This randomized controlled trial investigated the effects of pectoralis major MRM on breast pain and breast engorgement in breastfeeding mothers and on breast milk intake and sleep patterns in newborns (Figure 1). This study adhered to the CONSORT (Consolidated Standards of Reporting Trials) reporting guidelines [16].

## Methods

This study was approved by the In­stitutional Review Board of Eulji University (No. EU17-01). Informed consent was obtained from participants.

### Sample and sampling

**Figure 2.**
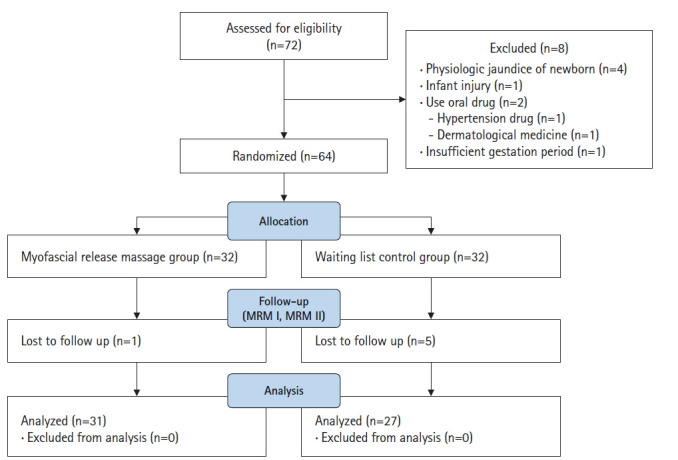
CONSORT flow diagram.

The participants were mothers at the postpartum care center affiliated with Sanbon Hospital in Gunpo, Korea. The inclusion criteria were delivery (vaginally or by cesarean section) of a newborn weighing at least 2,500 g between 37 to 42 weeks, without medication due to complications. Mothers who received breast massages at the hospital before entering the postpartum care center and mothers unable to directly breastfeed were excluded. Newborns with physiologic jaundice (total bilirubin, 12.0 mg/dL or higher) were also excluded. Considering the hospitalization period for delivery, mothers of newborns aged 7 to 14 days were recruited. The sample size was calculated using G*Power 3.1.9.2 (University of Düsseldorf, Düsseldorf, Germany) [17]. Parameters were chosen (effect size=.75; α=.05, and power=.80), and the t-test was selected as the method of analysis [18]; the results indicated a minimum requirement of 58 participants. In general, if the attrition rate is low (10%–15%), the sample is considered to be close to the target population [19]. Therefore, in this study, the probability of dropout rate was set at 10% and 64 participants were recruited. Of the 72 mothers originally identified as eligible, eight were excluded because they were unable to directly breastfeed due to physiological jaundice or neonatal injury, were taking medications, or were later found to have had a short gestation period. Random assignment was performed using the RAND function in Microsoft Excel (Microsoft Corp., Redmond, WA, USA) (Figure 2). Six women—one in the experimental group and five in the control group—were excluded from the study because they did not participate in the second session. Upon completion, the study included a total of 58 participants: 31 in the experimental group and 27 in the control group.

### Outcome measurements

#### Primary outcomes

##### 1) Breast pain

The degree of breast pain perceived by the participants was assessed using an 11-point scale from no breast pain (0 points) to extreme breast pain (10 points).

#### Secondary outcomes

##### 1) Breast engorgement

A rubber hardness tester (SHORE C; Yueqing Handpi Instruments Co., Ltd, Zhejiang, China) was used to measure changes in breast engorgement. Participants were placed in the supine position on a bed privately enclosed by a cubicle curtain. Then, a trained professional nurse examined the left and right breasts, and measurements were recorded in 1-point increments at the 2 o’clock and 10 o’clock positions, 3 cm from the nipples of both breasts.

##### 2) Breast milk intake of newborns

Breast milk intake was measured (g) before and after breastfeeding following the first MRM (MRM I) and the second MRM (MRM II) using a microscopic scale (CAS AD-15T; CAS Corp., Seoul, Korea).

##### 3) Formula supplementation

Formula supplementation was assessed by measuring the amount of formula supplement (mL) used over 48 hours by the breastfeeding mothers.

##### 4) Sleeping patterns of newborns

The total sleeping time of each newborn after breastfeeding was measured by the breastfeeding mother, who recorded the feeding type and sleeping pattern in a self-report questionnaire over 48 hours.

Most secondary outcomes were measured after both MRM I and MRM II; however, formula intake and sleep were measured only after MRM I, since many mothers had been discharged by the time at which the MRM II measurement would have been taken.

##### 5) General characteristics

The sociodemographic characteristics of breastfeeding mothers and newborns (e.g., maternal age, gestational age, nipple shape, breastfeeding education in pregnancy, delivery type, and neonatal age, height, weight, birth order, and sex of the newborn) were collected.

#### Experimental treatment

##### 1) Pectoralis major myofascial release massage 

**Figure 3.**
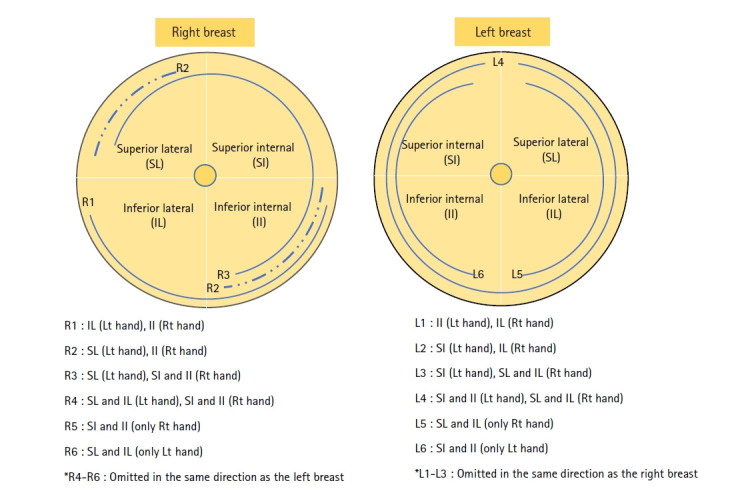
Myofascial release massage techniques. II: Inferior internal; IL: inferior lateral; Lt: left; Rt: right; SI: superior internal; SL: superior lateral.

The experimental treatment was performed by a nurse specializing in breast care, who had completed the International Board Certified Lactation Consultant course and had worked in a breast care counseling room for 10 years. MRM, a breast massage method that improves blood circulation in the breast and relieves pain caused by breast engorgement by releasing the pectoralis major muscle from the breast tissue in the chest, was provided in the breastfeeding care consultation room. This was a quiet area designated for the study, and it provided an environment that maintained personal privacy by enabling participants to wear a comfortable open gown while positioned behind curtains. With the participant supine on a bed, both breasts were massaged at 5-minute intervals for a total of 30 minutes (Figure 3, Supplementary Figure 1). A MRM Ⅱ was administered in the same manner 48 hours after the MRM Ⅰ to confirm the effect of repeated treatment, under the assumption that the effect decreased over time after experimental treatment. Since mothers receiving the same care at the postpartum care center were targeted, a second massage was performed 48 hours later to exclude exogenous variables.

### Data collection

Data were collected between March and June of 2017 according to the following procedures:

1) Recruitment flyers were posted at the postpartum care center affiliated with Sanbon Hospital in Gunpo, Korea.

2) After explaining the purpose, method, duration of participation, potential side effects and risk factors, benefits of participation, confidential treatment of personal information, and researcher contact information, written consent was obtained.

3) Consenting women were allocated into groups by 1:1 parallel random allocation using Excel random number generation. The research participants were not provided with information about their assigned groups, but the data collector had this information; as such, this was a single-blind study.

4) A preliminary survey was administered before the start of the experiment by distributing questionnaires to the members of both groups.

5) In the experimental group, after the newborns were weighed, the participants received their MRMⅠ therapy and subsequently breastfed their newborns. The left and right breasts were divided into four areas, and the pectoralis major muscle and breast tissue were separated with six hand movements each (right breast, R1 to R6; left breast, L1 to L6) (Figure 3). MRM was administered on the participant’s right side using the provider’s left and right hands. MRM on the right breast comprised six hand motions from R1 to R6 within 1 minute, which was continued for 5 minutes. This method was repeated on the left breast from L1 to L6. The therapy was repeated three times, alternating between the right and left breasts, for a total of 30 minutes (Supplementary Figure 1). For the control group, the mothers breastfed their infants after the newborns were weighed.

6) For both groups, breast pain, breast engorgement, and the breast milk intake of the newborns were measured after breastfeeding, and the mothers completed a 48-hour report on newborn sleep patterns.

7) The MRM Ⅱ was performed as described above 2 days (48 hours) after the MRM Ⅰ, meaning that the study participants received two therapy sessions.

### Data analysis

The collected data were analyzed using IBM SPSS Statistics ver. 24.0 (IBM Corp., Armonk, NY, USA). The general characteristics of the breastfeeding mothers and newborns were analyzed by frequency, percentage, mean, and standard deviation. The homogeneity of the two groups was verified using the t-test and the chi-square test. The normality of the data distribution for each variable was checked. The differences between the two groups in the degrees of breast pain and breast engorgement, the amount of breast milk intake of the newborns, and the sleeping patterns of the newborns were analyzed using the t-test. Breast pain, breast engorgement, and neonatal breast milk intake over time were analyzed with repeated-measures analysis of variance.

## Results

### Verification of the homogeneity of participants

Participants’ general characteristics and the dependent variables showed no statistically significant differences between the two groups; therefore, homogeneity was confirmed (Tables 1, 2).

### Effects of the pectoralis major myofascial release massage 

#### Breast pain

**Figure 4.**
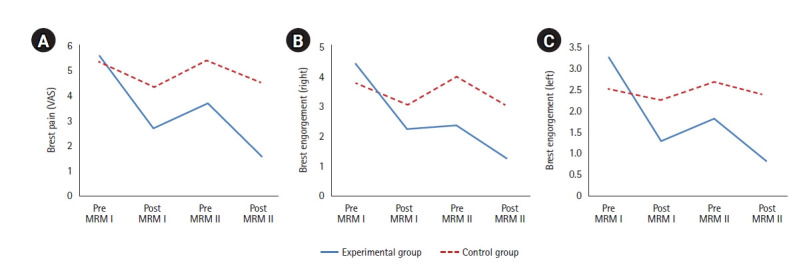
Results for breast pain and engorgement. MRM: Myofascial release massage; MRM I: the first pectoralis major MRM; MRM II: the second pectoralis MRM; VAS: visual analog scale.

No significant difference in breast pain was present between the two groups before experimental treatment; however, a significant difference was found in breast pain between groups after the MRM Ⅰ (t=−5.38, *p*<.001). Breast pain before the MRM Ⅱ was significantly lower in the experimental group than in the control group (t=−4.45, *p*<.001), and breast pain after the MRM Ⅱ was also significantly lower in the experimental group (t=−10.05, *p*<.001). The results of repeated-measures analysis of variance indicated a significant group-by-time interaction effect (F=27.57, *p*<.001) (Table 2, Figure 4).

#### Breast engorgement

The results for breast engorgement are shown in Table 2 and Figure 4. No significant difference in breast engorgement was present between the groups at baseline for either breast. However, after the MRM Ⅰ, the right breast received scores of 2.23 points and 3.07 points in the experimental group and control group, respectively, while the left breast received scores of 1.32 points and 2.56 points in the experimental group and control group, respectively. The latter difference was statistically significant (right: t=−1.68, *p*=.100; left: t=−2.13, *p*=.037). In other words, breast engorgement was not significantly different for the right breast after the MRM Ⅰ, but a significant difference was found for the left breast. A significant difference was noted for the right breast before the MRM Ⅱ, with 2.39 points measured in the experimental group and 4.00 points in the control group; however, no significant difference was observed for the left breast, which showed a score of 1.84 points in the experimental group and 2.70 points in the control group (right: t=−2.98, *p*=.005; left: t=−1.81, *p*=.075). After the MRM Ⅱ, a significant difference was seen in breast engorgement, with the right breast receiving a score of 1.23 points in the experimental group and 3.00 points in the control group, and the left breast receiving a score of 0.84 points in the experimental group and 2.41 points in the control group (right: t=−4.50, *p*<.001; left: t=−3.74, *p*<.001). A significant difference was found in the right breast before the MRM Ⅱ, but no significant difference was seen in the left breast. Following the MRM Ⅱ, the left and right breasts showed significantly less engorgement in the experimental group than in the control group. The interaction between time and group was also statistically significant (right; F=17.12, *p*<.001; left; F=14.10, *p*<.001). Overall, after the MRM intervention, breast engorgement was lower in the experimental group than in the control group. Softening of the breast was also greater in the experimental group than in the control group.

#### Newborn breast milk intake

Breast milk intake was measured twice by comparing the weights of the newborns before and after breastfeeding. The results are shown in Table 2. After the MRM Ⅰ, a significant difference was observed in newborns’ breast milk intake between the two groups (t=3.10, *p*=.003). A significant difference was also found in breast milk intake between the groups after the MRM Ⅱ (t=3.09, *p*=.003). That is, after both the first and second MRM interventions, newborns’ breast milk intake was higher in the MRM experimental group than in the control group.

#### Formula supplementation

The amount of infant formula supplementation used over the 48 hours following the MRM Ⅰ is shown in Table 2. After the MRM Ⅰ, a significant difference between the two groups was observed in the amount of infant formula supplementation used (t=−2.50, *p*=.015), with greater intake in the control group than in the experimental group.

#### Newborn sleeping patterns

No significant difference was noted in the newborns’ sleeping patterns during the 48-hour period after the MRM Ⅰ (t=0.38, *p*=.702).

## Discussion

This study found that MRM was effective in reducing breast pain and breast engorgement in breastfeeding mothers. These results align with a previous study reporting the alleviation of breast pain and reduction in breast engorgement after massaging the base of mothers’ breasts [10], as well as a study that reported effective relief of breast pain by fascia relaxation breast massage [18].

In the present study, MRM was applied twice over 48 hours. After MRM I, breast pain significantly decreased in the experimental group, and the effect continued for MRM II, again showing a significant difference between the two groups. After MRM II, breast pain was significantly reduced relative to before the MRM I treatment. Although direct comparison is limited, in a study [20] where women received general breast massages and cabbage therapy for 3 days after childbirth, breast pain was slightly reduced in the experimental group compared to the control group. Breast pain is caused by fascia tension, nerve entrapment and blood vessel constriction in the chest muscles; this aligns with the results of this study, since the pectoralis muscle massage was effective for immediate relief of breast pain by relaxing the breast muscles [15].

In this study, the right breast showed greater engorgement than the left breast. Following MRM intervention, breast engorgement significantly decreased. Like breast pain, in the previous study [20], breast engorgement was reduced after general breast massage therapy; however, in the present study, the average reductions in breast engorgement for both breasts were 2.85 and 0.47 points in the experimental group and control group, respectively. These results show a six-fold difference in breast engorgement reduction between the groups, demonstrating the effectiveness of the MRM intervention.

No prior studies have directly measured the breast milk intake of newborns after the application of breast massage therapy. Importantly, in this study, the amount of breast milk intake was directly measured using an objective indicator: the weight of newborns before and after breastfeeding. In the preliminary examination before the commencement of the study, the amount of milk powder intake and the weight before and after formula intake were confirmed to be consistent. Regarding weight gain, newborns in the experimental group gained 17 g more (approximately twice as much) than the newborns in the control group, a significant difference. Furthermore, insufficient breast milk intake was evaluated based on the amount of formula supplementation consumed. The amount of formula consumed over the 48 hours after the MRM Ⅰ was significantly lower in the experimental group than in the control group. Therefore, MRM was effective in increasing the amount of breast milk consumed by the newborns.

The sleeping patterns of newborns are affected by various sleep environment factors. However, in this study, sleeping time was evaluated to assess the specific effects of differences in breast milk intake on sleeping patterns after breastfeeding. The average total sleeping time of the newborns in both groups was 19 hours per day, with no significant difference observed between the groups in total sleeping time after the MRM Ⅰ. This is consistent with a previous study [21].

This study focused on breastfeeding mothers and their newborns and examined the effects of pectoralis major MRM on engorgement, breast pain, breast milk intake, formula supplementation, and newborns’ sleeping patterns by employing direct measurement methods. To generalize pectoralis major MRM as a nursing intervention to improve the rate of breastfeeding practice, studies of various types with diverse participant groups are necessary. Moreover, the continuity of the effect must be evaluated by examining the rate of breastfeeding practice and the type of feeding after 6 months.

A limitation of the study is that since it was conducted at a postpartum care center affiliated with a hospital, its generalizability may be limited. Furthermore, although the study gathered data on the delivery type (cesarean vs. vaginal delivery), differences according to delivery type were not found. Finally, as only the effects of MRM were investigated, a more comprehensive comparison with other breast care interventions would be helpful in the future.

In conclusion, this study revealed that MRM delivered to breastfeeding mothers in two 30-minute sessions separated by a 48-hour interval was effective in reducing breast pain, reducing the severity of breast engorgement, and increasing breastfeeding in newborns. In particular, a significant difference was observed in breast pain between the experimental and control groups following the first and second MRM sessions, and a significant group-by-time interaction effect was noted. Since pectoralis major MRM is effective and relatively easy to administer in clinical practice, it can be utilized as a nursing intervention to alleviate discomfort during breastfeeding and increase newborns’ breast milk intake. The active application of MRM may consequently improve the continuation of breastfeeding.

## Figures and Tables

**Table 1.** Homogeneity test of general characteristics (N=58)

Characteristic	Categories	Mean±SD or n (%)		χ^2^or t (*p*)
		Exp (n=31)	Cont (n=27)	
Age (year)		32.13±3.02	33.63±3.19	−1.84 (.071)
Gestational age (day)		276.16±7.92	275.30±6.44	0.45 (.653)
Newborn age (day)		8.90±2.21	8.00±1.69	1.76 (.084)
Newborn height (cm)		50.73±2.42	51.28±1.35	−1.09 (.281)
Newborn weight at birth (g)		3,252.58±491.57	3,371.11±277.87	−1.15 (.256)
Birth order	First	25 (80.6)	20 (74.1)	0.36 (.549)
	Second or later	6 (19.4)	7 (25.9)	
Type of delivery	Normal	23 (74.2)	18 (66.7)	0.40 (.530)
	Cesarean	8 (25.8)	9 (33.3)	
Newborn sex	Male	13 (41.9)	12 (44.4)	0.37 (.847)
	Female	18 (58.1)	15 (55.6)	
Nipple shape	Normal	11 (35.5)	10 (37.0)	4.60 (.100)
	Flat	13 (41.9)	16 (59.3)	
	Inverted	7 (22.6)	1 (3.7)	
Breastfeeding education during pregnancy	Yes	4 (12.9)	5 (18.5)	0.35 (.556)
	No	27 (87.1)	22 (81.5)	

Cont: Control group; Exp: experimental group.

**Table 2.** Comparison of breast pain and breast engorgement between groups (N=58)

Characteristic	Mean±SD		t	*p*	F(p)^*^
	Exp (n=31)	Cont (n=27)			
Breast pain (VAS)					
Pre-MRM I	5.61±1.15	5.37±1.57	0.68	.501	T: 91.79 (*p*<.001)
Post-MRM I	2.71±0.94	4.33±1.30	−5.38	<.001	T*G: 27.57 (*p*<.001)
Pre-MRM II	3.71±1.37	5.44±1.60	−4.45	<.001	G: 34.54 (*p*<.001)
Post-MRM II	1.58±0.77	4.52±1.34	−10.05	<.001	
Breast engorgement (right)					
Pre-MRM I	4.45±1.95	3.78±2.52	1.15	.256	T: 59.93 (*p*<.001)
Post-MRM I	2.23±1.56	3.07±2.27	−1.68	.100	T*G: 17.12 (*p*<.001)
Pre-MRM II	2.39±1.36	4.00±2.51	−2.98	.005	G: 3.59 (*p*=.063)
Post-MRM II	1.23±1.09	3.00±1.86	−4.50	<.001	
Breast engorgement (left)					
Pre-MRM I	3.32±1.83	2.56±2.14	1.47	.147	T: 27.11 (*p*<.001)
Post-MRM I	1.32±1.30	2.56±2.01	−2.13	.037	T*G: 14.10 (*p*<.001)
Pre-MRM II	1.84±1.49	2.70±2.13	−1.81	.075	G: 2.49 (*p*=.120)
Post-MRM II	0.84±0.97	2.41±1.99	−3.74	<.001	
Newborn’s breast milk intake (g)					
Post-MRM I	35.16±25.02	17.78±17.39	3.10	.003	
Post-MRM II	36.13±22.01	18.89±20.25	3.09	.003	
Formula supplementation intake over 2 days (mL)					
Post-MRM I	334.84±270.76	530.74±325.16	−2.50	.015	
Total sleep time over 2 days					
Post-MRM I	2,295.71±273.80	2,265.19±330.89	0.38	.702	

Cont: Control group; Exp: experimental group; MRM: major myofascial release massage; MRM I: the first pectoralis MRM; MRM II: the second pectoralis MRM; VAS: visual analog scale.

* Repeated-measures analysis of variance.

## Supplementary materials

Further details on supplementary materials are presented online (available at https://doi.org/10.4069/kjwhn.2023.03.15).

Supplementary Figure 1. Six steps of myofascial release massage.
