# Corrigendum: Effect of pectoralis major myofascial release massage for breastfeeding mothers on breast pain, engorgement, and newborns’ breast milk intake and sleeping patterns in Korea: a randomized controlled trial

**DOI:** 10.4069/kjwhn.2023.03.15.e1

**Published:** 2023-06-19

**Authors:** Won-Ryung Choi, Yeon-Suk Kim, Ju-Ri Kim, Myung-Haeng Hur

**Affiliations:** 1 Sanbon Branch of Mamslibe, Gunpo, Korea; 2 Department of Nursing, Chung Cheong University, Cheongju, Korea; 3 Department of Nursing, Kyung Min University, Uijeongbu, Korea; 4 College of Nursing, Eulji University, Uijeongbu, Korea

Korean J Women Health Nurs 2023;29(1):66-75.

https://doi.org/10.4069/kjwhn.2023.03.15

This corrigendum is for correcting a reference that was mistakenly reported in the above article.

4. Pereira-Kotze C, Feeley A, Doherty T, Faber M. Maternity protection entitlements for non-standard workers in low-and-middle-income countries and potential implications for breastfeeding practices: a scoping review of research since 2000. Int Breastfeed J. 2023;18(1):9. https://doi.org/10.1186/s13006-023-00542-8

The corrected reference is as below. The authors apologize for any inconvenience that this may have caused.

4. Brandhagen M, Lissner L, Brantsaeter AL, Meltzer HM, Häggkvist AP, Haugen M, et al. Breast-feeding in relation to weight retention up to 36 months postpartum in the Norwegian Mother and Child Cohort Study: modification by socio-economic status? Public Health Nutr. 2014;17(7):1514-1523. https://doi.org/10.1017/s1368980013001869.

