# Supplementary material for: A randomized controlled trial of pectoralis major myofascial release massage for breastfeeding mothers: breast pain, engorgement, and newborns’ breast milk intake and sleeping patterns
Source: Korean J Women Health Nurs. 2023 Mar 31;29(1):66–75. doi: 10.4069/kjwhn.2023.03.15 (PMC10085664; doi:10.4069/kjwhn.2023.03.15)
Supplement: Supplementary Figure 1. — Six steps of myofascial release massage. [file kjwhn-2023-03-15-Supplementary-Figure-1.pdf]

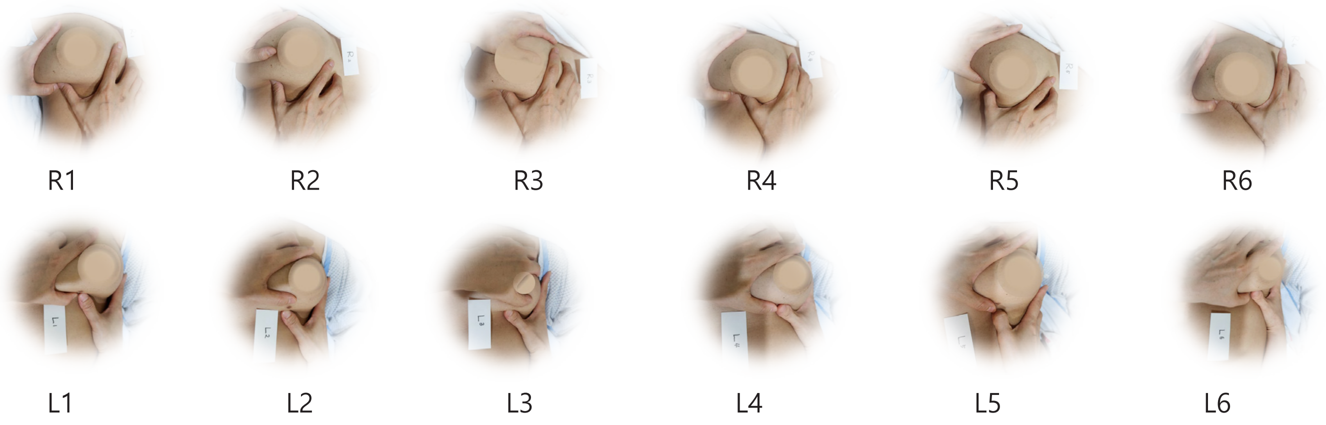

Myofascial release massage consists of 6 steps.

Myofascial release massage is the release of myofascial muscles, which focuses on stretching and applying manual pressure to allow more freedom and gentleness of limited movement of the muscles, and providing rubbing of the muscles to make the movements smooth and painless.

Supplementary Figure 1. Six steps of myofascial release massage.
